# Targeted long-read sequencing identifies missing pathogenic variants in unsolved Werner syndrome cases

**DOI:** 10.1136/jmedgenet-2022-108485

**Published:** 2022-05-09

**Authors:** Danny E. Miller, Lin Lee, Miranda Galey, Renuka Kandhaya-Pillai, Marc Tischkowitz, Deepak Amalnath, Avadh Vithlani, Koutaro Yokote, Hisaya Kato, Yoshiro Maezawa, Aki Takada-Watanabe, Minoru Takemoto, George M. Martin, Evan E. Eichler, Fuki M. Hisama, Junko Oshima

**Affiliations:** 1 Department of Pediatrics, Division of Genetic Medicine, University of Washington, Seattle, Washington, USA; 2 Department of Genome Sciences, University of Washington School of Medicine, Seattle, Washington, USA; 3 Department of Laboratory Medicine and Pathology, University of Washington, Seattle, Washington, USA; 4 Department of Medical Genetics, National Institute for Health Research Cambridge Biomedical Research Centre, University of Cambridge, Cambridge, UK; 5 Department of Medicine, Jawaharlal Institute of Postgraduate Medical Education and Research, Puducherry, India; 6 Department of Endocrinology, Hematology and Gerontology, Chiba University Graduate School of Medicine, Chiba, Japan; 7 Department of Diabetes, Metabolism and Endocrinology, International University of Health and Welfare, Otawara, Japan; 8 Howard Hughes Medical Institute, University of Washington, Seattle, Washington, USA; 9 Department of Medicine, Division of Medical Genetics, University of Washington, Seattle, Washington, USA

**Keywords:** nanopore sequencing, genomics, genetic variation

## Abstract

**Background:**

Werner syndrome (WS) is an autosomal recessive progeroid syndrome caused by variants in *WRN*. The International Registry of Werner Syndrome has identified biallelic pathogenic variants in 179/188 cases of classical WS. In the remaining nine cases, only one heterozygous pathogenic variant has been identified.

**Methods:**

Targeted long-read sequencing (T-LRS) on an Oxford Nanopore platform was used to search for a second pathogenic variant in *WRN*. Previously, T-LRS was successfully used to identify missing variants and analyse complex rearrangements.

**Results:**

We identified a second pathogenic variant in eight of nine unsolved WS cases. In five cases, T-LRS identified intronic splice variants that were confirmed by either RT-PCR or exon trapping to affect splicing; in one case, T-LRS identified a 339 kbp deletion, and in two cases, pathogenic missense variants. Phasing of long reads predicted all newly identified variants were on a different haplotype than the previously known variant. Finally, in one case, RT-PCR previously identified skipping of exon 20; however, T-LRS did not detect a pathogenic DNA sequence variant.

**Conclusion:**

T-LRS is an effective method for identifying missing pathogenic variants. Although limitations with computational prediction algorithms can hinder the interpretation of variants, T-LRS is particularly effective in identifying intronic variants.

**Key messages**

**What is already known on this topic**

- The burden of undiagnosed genetic disorders on individuals is high.
- In some cases, a specific genetic disorder is suspected but no variants are found in genes associated with that disorder.
- In other cases, testing identifies a single variant in a gene associated with the suspected disorder but no second variant.

**What this study adds**

- This study reports the results of targeted long-read sequencing (T-LRS) in a cohort of individuals highly suspected to have a specific genetic disorder, Werner syndrome, but only one pathogenic variant identified in the causative gene, *WRN*.
- This study demonstrates that T-LRS in a well-phenotyped cohort has a high yield.

**How this study might affect research, practice or policy**

- The implications of this work are that in a well-defined cohort with a clear phenotype T-LRS represents an excellent next best test after non-diagnostic clinical testing.

## Introduction

Segmental progeroid syndromes are a group of disorders that phenotypically resemble accelerated ageing.^1^ The prototypical example of an adult-onset progeroid syndrome is Werner syndrome (WS; OMIM #277700), a rare autosomal recessive disorder caused by loss-of-function variants of the gene *WRN*.^2 3^ Individuals with WS typically do not show clinical signs until their early teens when the first clinical sign—lack of a growth spurt (often recognised retrospectively)—appears. An aged appearance (grey hair, atrophic skin) begins to develop in the 20s and 30s, which is followed by a series of common age-related diseases, including bilateral cataracts, gonadal atrophy, type II diabetes mellitus, osteoporosis and arteriosclerosis.^3 4^ Alzheimer-type dementia is generally not a feature of WS. The most common causes of death in WS are myocardial infarction and malignancies at a median age of 54 years.^5 6^ The most significant quality-of-life issue is development of deep ulcerations around the ankles and, occasionally, the elbows, which are associated with soft tissue calcifications and may eventually require lower limb amputation.^7^

*WRN* encodes a multifunctional nuclear protein with exonuclease and RecQ-type helicase domains.^4^ Molecular and cellular evidence suggest the involvement of *WRN* in a wide variety of functions, including DNA repair, recombination, replication and telomere maintenance.^4^ Cells derived from individuals with WS exhibit limited replicative lifespan, altered epigenetic signatures and mitochondrial dysfunction.^4 8 9^ More recently, *WRN* helicase activity was shown to be essential for the survival of mismatch repair-deficient cancer cell lines, suggesting some functional overlap of these DNA repair pathways.^10^

To date, nearly 100 different pathogenic variants have been reported in individuals with WS worldwide.^2 11^ The majority of disease-causing variants in WS result in truncation of the WRN protein and the elimination of the nuclear localisation signal at the C-terminus and/or nonsense-mediated mRNA decay, making them functionally null.^2^ That most variants result in little to no protein expression seems to be why all individuals with WS share similar phenotypes regardless of the causal variants. Several amino acid substitutions that abolish helicase activities have also been identified but only as compound heterozygous variants in combination with null variants.^12^ Founder variants have been detected in specific populations, such as in Sardinia and Japan where carrier frequencies as high as 1:150 have been observed for specific variants.^2 13^ Possible founder mutations have also been reported in India, Pakistan, Turkey and the Netherlands.^14 15^ Differences in WS presentations among various populations have also been reported. For example, Indian/Pakistani individuals with WS tend to have earlier onset of cataracts, at a median age of 20 years compared with 31 years in non-Indian individuals with WS.^14^

Established in 1988, the International Registry of Werner Syndrome at the University of Washington recruits individuals with suspected progeroid syndromes from all over the world for molecular diagnosis and further biological study. Potential therapeutic approaches are being sought out in collaboration with the Japanese Werner Consortium.^8 16 17^ As of November 2021, the Registry has enrolled 179 individuals with classical WS with documented biallelic causal variants and 9 individuals with classical WS presentations in which only a single heterozygous causal variant has been identified. Recently, targeted long-read sequencing (T-LRS) on the Oxford Nanopore Technologies (ONT) platform was used to clarify complex structural variants and identify missing variants in cases that remained unsolved despite a complete clinical evaluation.^18^ We hypothesised that T-LRS could identify a second pathogenic variant in the unsolved WS cases. Of the nine molecularly unsolved cases from eight pedigrees in the registry, we identified a second pathogenic variant in eight. A second pathogenic variant was not identified in one case with known skipping of exon 20, despite long-read sequencing and phasing, demonstrating the limitation of both T-LRS and currently available prediction algorithms used to interpret DNA variants.

## Materials and methods

### Recruitment of study participants, sample processing and standard sequencing analysis

Individuals are referred to the International Registry of Werner Syndrome at the University of Washington by physicians who suspect a diagnosis of WS. Biological samples collected from individuals suspected to have WS who consent to be enrolled in the study are shipped to the International Registry.

Blood and skin sample processing was performed as previously described.^5^ Briefly, participant blood samples were processed immediately on arrival for cryopreservation of primary cells and plasma, isolation of DNA and RNA and establishment of lymphoblastoid cell lines (LCLs) using Epstein-Barr virus. Depending on the year of referral, different methods of DNA and RNA sequencing were done (table 1). In some cases, Sanger sequencing of some exons was done, while in others, sequencing was done on an exome backbone.^2 5 15 19^ RT-PCR sequencing was performed on total RNA isolated from blood or LCLs. Western blot analysis was done using total protein isolated from LCLs. Detailed protocols for Sanger sequencing and western blot analyses have been previously described.^2 5 19^

**Table 1:** Targeted long-read sequencing (T-LRS) identified candidate pathogenic variants in eight of nine individuals with Werner syndrome (BIA1010 and BIA1020 are siblings)

Registry	Known variant (all heterozygous)	Additional workup	T-LRS result	Confirmation of T-LRS result
WV	RT-PCR: c.2773delG, p.A925fs	Western: no protein	c.3961C>T, p.R1321*,	PCR+Sanger
BIA1010	Sanger: c.1105C>T, p.R369*	Western: no protein	c.3234-170A>G	RT-PCR
BIA1020	Sanger: c.1105C>T, p.R369*	Western: no protein	c.3234-170A>G	RT-PCR
PD1010	Exome sequencing: c.561A>G, p.K187*	Western: no protein	chr8:g.31135822_ 31 474 535delinsTCT	PCR+Sanger
CB4	Sanger: c.3139-1G>C, p.G1047fs	Western: no protein SNP array: normal	c.1982-297A>G	RT-PCR
CB6	Sanger: c.1105C>T, p.R369*	Western: no protein SNP array: normal	c.1982-297A>G	RT-PCR
FES	RT-PCR: c.1165delA, p.R389fs	Western: no protein	c.2103_2104delAC, p.Leu702fs	PCR+Sanger
SILV1010	Sanger: c.2665C>T, p.R889*	Western: ~1% protein RT-PCR: skipping of exon 20	No second variant found	n/a
EN1010	Genome sequencing: c.2367_2368delAT, p.S790fs	None	c.839+1309T>G	Exon trap (figure 3)

n/a, not available.

### Targeted long-read sequencing of the *WRN* locus

T-LRS was performed using ReadFish^20^ on an ONT GridION as previously described.^18^ Briefly, for each sample 1–2 µg of genomic DNA was sheared using a Covaris g-TUBE by centrifuging at 6000 rpm for 2 min then inverting and centrifuging at 6000 rpm again for 2 min. DNA was prepared for sequencing using the ONT Ligation Kit (SQK-LSK109) following the manufacturer’s instructions. Each library was loaded onto one or more R9.4.1 flow cells (FLO-MIN106D) and run for 72 hours with the goal of recovering at least 20× coverage per library (online supplemental table 1). For each sample, an approximately 3 Mbp region surrounding *WRN* was targeted along with two control regions (online supplemental table 2).

10.1136/jmedgenet-2022-108485.supp1 Supplementary data

### Long-read sequencing of RT-PCR products from individual SILV1010

To identify a second pathogenic variant in SILV1010, we performed overlapping RT-PCR of *WRN* mRNA using previously published primers.^19^ The RT-PCR product was prepared for ONT sequencing using the SQK-LSK109 ligation kit following the manufacturer’s instructions. A single library was loaded onto a Nanopore R9.4.1 Flongle flow cell (FLO-FLG001) and sequenced for 24 hours. Reads were aligned to GRCh38 using minimap2.^21^

### Data analysis

Fast5 files were base called using the high accuracy model in Guppy 4.0.11 (ONT). Given no second candidate pathogenic variant was identified in SILV1010, it was re-base called using the superior model in Guppy 5.0.7 (ONT). For all samples, all FASTQ reads were aligned to GRCh38 using minimap2.^21^ Single-nucleotide and insertion/deletion variants (SNVs and indels) were called with Medaka (ONT), which was also used for phasing. Variant calling and phasing were redone using Guppy 5 data for SILV1010 using Clair3.^22^ Structural variants were identified using Sniffles,^23^ SVIM^24^ and CuteSV.^25^ VEP V.103.1^26^ was used to annotate VCF files produced by Guppy or Medaka and add splice predictions from SpliceAI,^27^ CADD V.1.8 scores^28^ and population allele frequency from the gnomAD V.2.1.1 genomes file.^29^ Aligned reads were visualised using integrative genomics viewer.^30^ Analysis was performed by filtering the VCF file by allele frequency and examining variants absent from population databases or those with allele frequencies <1%.

### Exon trapping assay

The 1.8 kbp region that spans intron 7 to intron 8 (c.724-167 to c.839+1553) was amplified from genomic DNA of individual EN1010 and subcloned into the multicloning sites of the pSPL3 vector^31 32^ linearized with EcoRI and PstI, using the NEBuilder HiFi DNA assembly cloning kit (catalogue# E5520, New England Biolabs) to generate pSPL3-EN1010-Wt and pSPL3-EN1010-Mut. These constructs were transfected to immortalised control human fibroblasts, 82-6hTERT, using FuGENE 6 (catalogue# E2693, Promega). After 48 hours, total RNA was isolated and reverse-transcribed with High-Capacity cDNA Reverse Transcription Kit (catalogue# 4368814, Thermo Fisher Scientific). RT-PCR was done using primers: SD6 (5'-TCTGAGTCACCTGGACAACC-3'), SA2 (5'-ATCTCAGTGGTATTTGTGAGC-3') and one designed from *WRN* exon 8, J20 (5'-CTGAGGAAGTGATGGATCTGG-3').

## Results

### Individuals sequenced in this report

Seven of the nine individuals with a single heterozygous variant in *WRN* have been previously described.^2^ All nine individuals were referred to the Registry with a clinical diagnosis of WS (table 2), two of which are newly described below. Variants in *WRN* were initially identified by Sanger sequencing of coding exons, which, over time, was gradually replaced by clinical exome or genome sequencing. A western blot analysis for WRN was done in cases for which material was available. RT-PCR and SNP arrays were also used in selected cases.^2 5 15^

**Table 2:** Clinical signs of Werner syndrome in seven previously reported cases

Registry#	EN1010	PD1010	BIA1010	BIA1020	SILV1010	CB4	WV
Age at evaluation (years)	42	42	45	38	68	40s	40
Cardinal signs							
Cataracts	Y	Y	Y	Y	Y*	Y	Y
Skin and facial feature	Y	Y	Y	Y	N	Y	Y
Short stature	N	Y	Y	Y	N	Y	Y
Greying or loss of hair	Y	Y	Y	Y	Y	Y	Y
Parental consanguinity or affected sibs	N	N	Y	Y	Y	N	NA
Further signs and symptoms							
Diabetes mellitus	N	Y	Y	Y	Y	Y	N
Hypogonadism	N	NA	Y	Y	NA	NA	Y
Osteoporosis	Y	Y	Y	Y	NA	NA	Y
Osteosclerosis	N	NA	Y	NA	NA	NA	NA
Soft tissue calcification	N	N	Y	Y	Y	Y	Y
Atherosclerosis	N	NA	Y	NA	NA	Y	NA
Neoplasms	N	NA	Y	NA	Y	N	NA
Voice change	Y	Y	Y	Y	Y	Y	Y
Flat feet	N	N	Y	Y	Y	Y	NA
Werner syndrome diagnostic criteria	Possible	Possible	Definite	Definite	Possible	Probable	Probable

*Unilateral cataract.

N, no; NA, not available; Y, yes.

Individual EN1010 is a man in his 50s born to non-consanguineous parents with a birth weight of 2.7 kg. He began to experience signs of premature ageing in his 20s with greying hair. He underwent bilateral cataract surgery in his early 30s and was subsequently diagnosed with hypothyroidism and type 2 diabetes mellitus in his 30s. He was diagnosed with hypogonadism and a prolactinoma in his 40s. His other medical history includes cardiac atherosclerosis, valve insufficiency and tinnitus.

On physical examination, his body mass index (BMI) was 20.2 kg/m^2^, height of 168 cm (Z-score −1.2) and weight of 57 kg (Z-score of −1.34). He had a mildly elevated blood pressure (148/62) and appeared progeroid, with grey hair, vocal cord atrophy, tight, atrophic skin with a waxy appearance and ulcers of the right heel and left metatarsal. His limbs were thin and notable for cold fingers, reduced subcutaneous fat, muscle atrophy and flat feet. Dual-energy X-ray absorptiometry scan revealed osteoporosis, osteosclerosis of the fingers and toes and soft tissue calcification. He had elevated liver enzymes with hepatic steatosis observed on abdominal MRI.

His family history includes two unaffected siblings: a sister in her 40s who is 160 cm tall (Z-score −0.5) and a brother in his 50s who is 170 cm tall (Z-score −0.92); each has one child. The father is in his 80s; his height is 180 cm (Z-score 0.48). The mother is in her 70s; her height is 160 cm (Z-score −0.5) and she has hypothyroidism and a pacemaker. Although he lacked short stature, this individual had three cardinal signs of WS (cataracts, dermatological pathology and premature greying) as well as additional clinical features, including type 2 diabetes mellitus and atherosclerosis; thus, this individual met criteria for possible diagnosis of WS diagnosis.^3^ Genome sequencing revealed a novel heterozygous *WRN* variant, c.2367_2368delAT, in exon 20, which results in the truncation of the WRN protein, p.Ser790fs (table 1). A second pathogenic variant was not reported.

Individual PD1010 is a South Asian man in his 40s born to non-consanguineous parents who was diagnosed with Addison disease in his teens, hypothyroidism and type 2 diabetes mellitus in his 20s (which later became insulin-dependent) and bilateral cataracts requiring surgery in his 30’s. On physical examination, his BMI was 17.8 kg/m^2^, height was 150 cm (Z-score −3.54) and weight was 40 kg (Z-score −4.3). He had a progeroid appearance, with sparse grey hair, a high-pitched squeaky voice and atrophic, thin skin. His limbs were thin, with loss of subcutaneous fat, muscle wasting and ulcers at both elbows. He had no siblings.

Exome sequencing revealed a heterozygous variant in exon 6 of *WRN*, c.561A>G (table 1). This synonymous variant is a known founder mutation seen in individuals of Indian or Pakistani descent and has been shown to activate a cryptic splice site in exon 6, resulting in p.Lys187fs.^14^ A second pathogenic variant was not identified by exome sequencing. Western blot analysis of protein from LCLs showed no detectable WRN protein, confirming the diagnosis of WS.

### Detection of deep intronic splice variants by T-LRS

**Figure 1:**
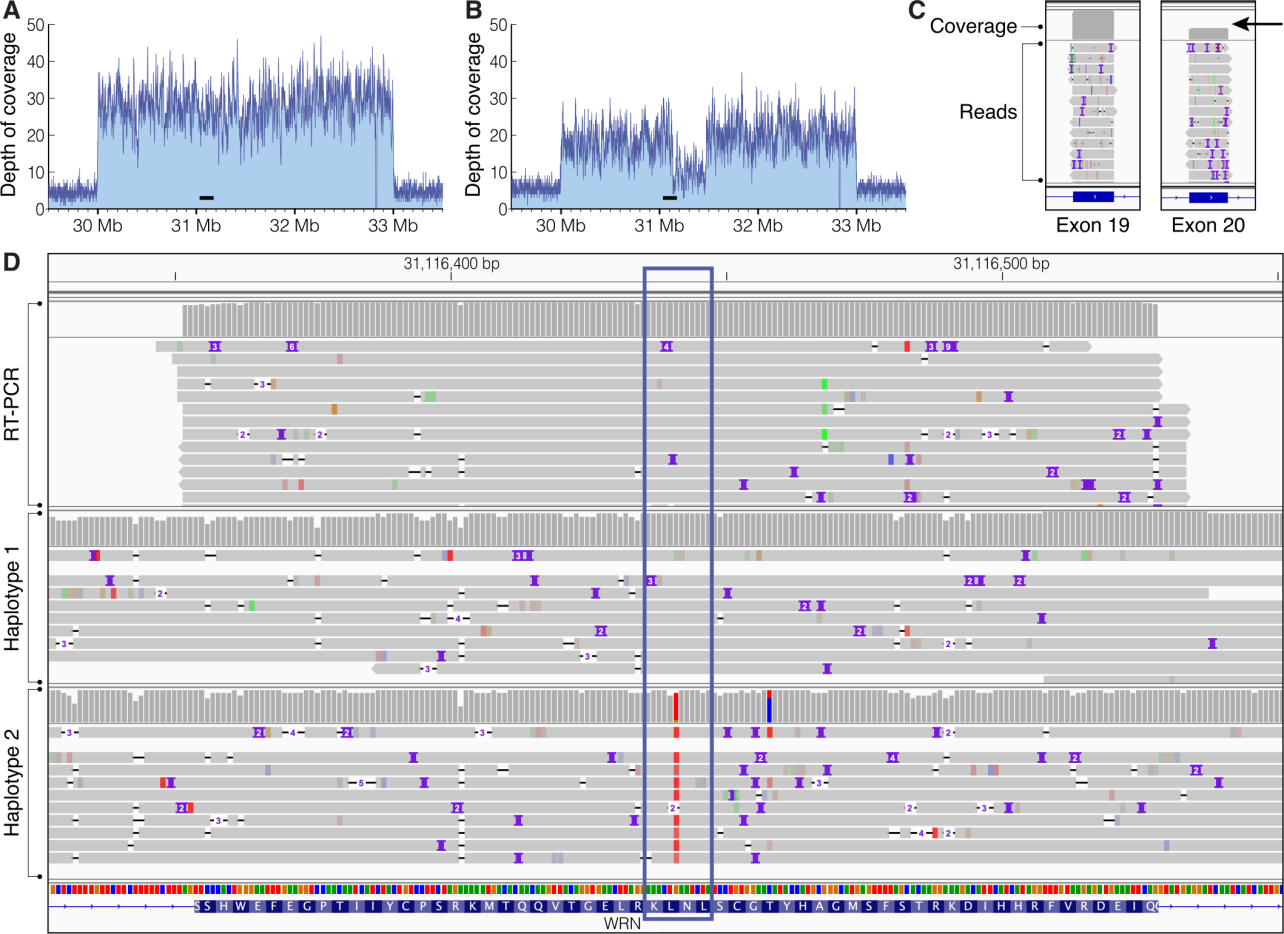
Targeted long-read sequencing (T-LRS) was used to identify missing variants. (A) T-LRS was used to target an approximately 3 Mbp region around *WRN*. Data shown for individual WV. Increased coverage (y-axis) represents the target region, decreased coverage represents background. The position of *WRN* is represented by the horizontal black bar around 31 Mbp (GRCh38). (B) T-LRS revealed a 339 kbp deletion in individual PD1010 that began within *WRN*. (C) Long-read sequencing of RT-PCR products from individual SIV1010 confirmed the absence of exon 20 in approximately half of the reads (arrow). Please see online supplemental figure 3 for an integrative genomics viewer screenshot of exons 19–21 that shows how reads are linked and that approximately half of reads skip of exon 20. (D) Long-read sequencing revealed RT-PCR products from individual SIV1010 did not carry the T allele present on haplotype 2 within exon 20, confirming that splicing was altered on haplotype 2. The previously known pathogenic variant (table 1) was predicted to be located on haplotype 1. No second pathogenic variant was identified in this individual.

**Figure 2:**
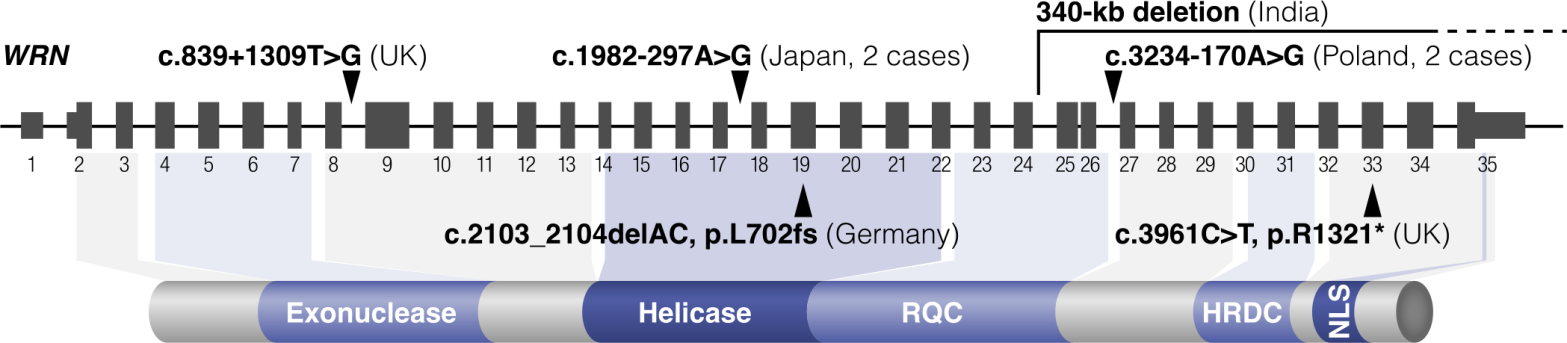
Diagram of *WRN* and the location of variants identified in this study. Pathogenic variants found in this study are shown with respect to the 35 exons within *WRN*. Functional domains of WRN proteins are shown below the corresponding exons. Those functional domains are the exonuclease and helicase domains, RecQ helicase conserved region (RQC), helicase RNaseD C-terminal conserved region (HRDC) and the nuclear localisation signal (NLS).

We performed T-LRS on eight of the nine molecularly unsolved cases with an average depth of coverage of 20× (figure 1A, online supplemental table 1). In all eight sequenced cases, the known pathogenic variant was identified. In four of the eight sequenced cases, T-LRS identified an intronic variant predicted by SpliceAI^27^ to alter splicing (figure 2, table 1, online supplemental table 3). One individual was not sequenced because a splice variant was found in his affected brother (BIA1010 and BIA1020). In all four cases, phasing predicted that the intronic splice variant is on a different haplotype than the previously identified pathogenic variant.

Individuals BIA1010 and BIA1020 were affected siblings from Poland carrying a heterozygous c.1105C>T, p.Arg369*, a known founder variant, accounting for approximately 20% of the mutant alleles in our registry.^2^ T-LRS revealed a heterozygous c.3234-170A>G in intron 26, which was predicted by SpliceAI to create a cryptic splice acceptor site (AA→AG). RT-PCR of the region including exons 25 and 26 showed two abnormal transcripts, one with a 69 bp insertion (r.3233_3234ins69) and the other with a 169 bp insertion (r.3233_3234ins169) between exons 25 and 26 (online supplemental figure 1). Both cryptic exons started at c.3234-169, as predicted by in silico analysis. Within the family, PCR and Sanger sequencing confirmed that the two variants were from the parents: c.3234-170A>G was transmitted from the mother (BIA1040) and c.1105C>T from the father (BIA1050).

In two unrelated Japanese individuals with WS, CB4 and CB6, T-LRS detected a c.1982-297A>G in intron 17. In silico analysis predicted that this substitution activates the adjacent GT to be the cryptic splice donor (A|GT→G|GT). RT-PCR sequencing revealed the presence of the corresponding 73 bp cryptic exon between exons 17 and 18 (r.1981_1982ins73) in CB6. RT-PCR was not done for individual CB4 because there was insufficient remaining mRNA.

**Figure 3:**
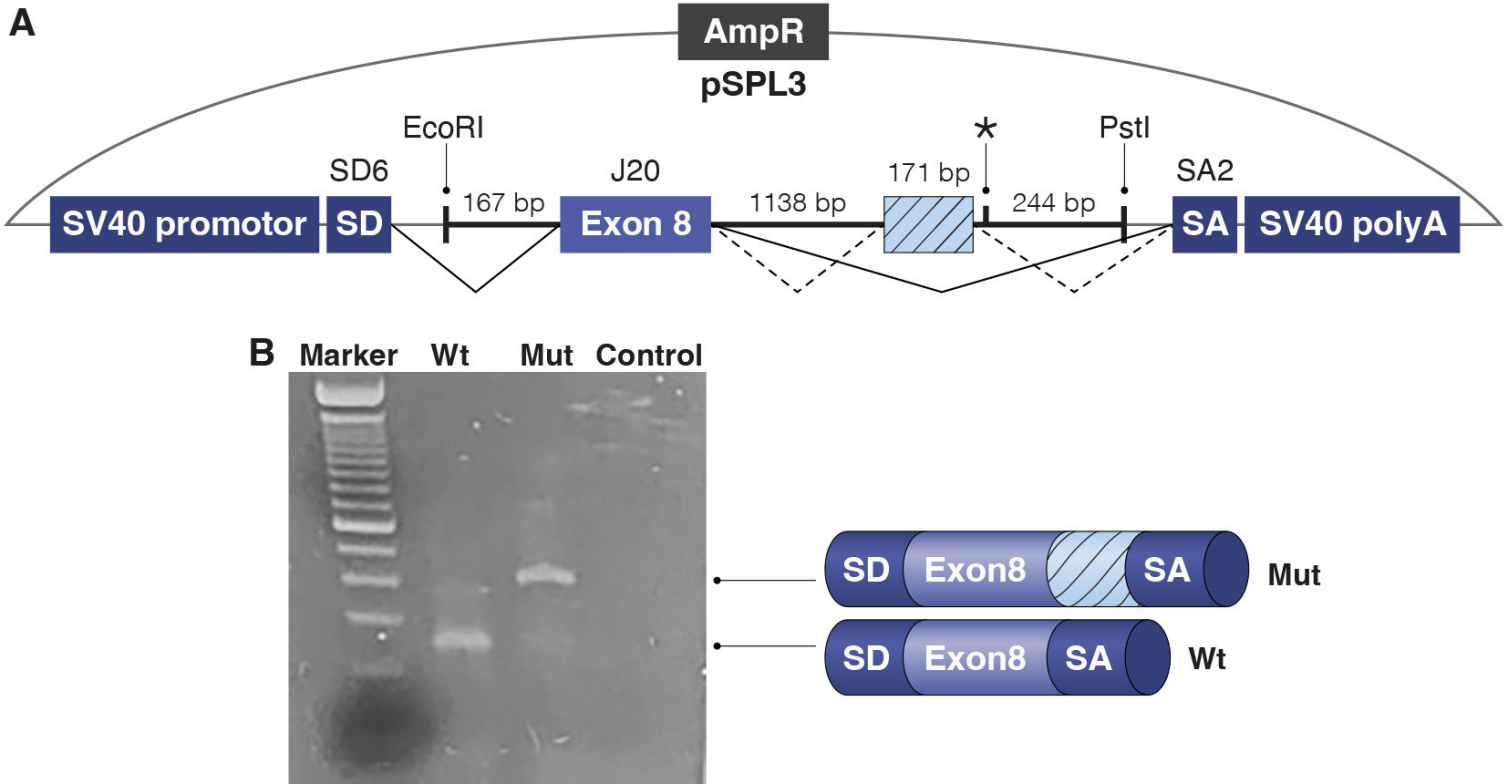
Exon trapping of the EN1010 variant. (A) The pSPL3 vector contains splice donor (SD) and splice acceptor (SA) exons and functional introns, with transcription beginning following the SV40 promoter and ending at the poly(A) site. The wildtype (Wt) construct, pSPL3-EN1010-Wt, and the mutant (Mut) construct, pSPL3-EN1010-Mut, contain the 1.8 kbp fragment derived from EN1010, with the c.839+1309T>G variant at the asterisk (*). (B) Agarose gel electrophoresis of RT-PCR products shows the larger amplicon in the Mut compared with Wt due to the 171 bp cryptic insertion.

For individual EN1010, T-LRS detected a c.839+1309T>G in intron 8, along with the previously identified c.2367_2368delAT in exon 20. This intron 8 variant has a CADD of 9.3 and SpliceAI predicts it creates a new donor site (T|GT→G|GT) with a score of 0.73. Unfortunately, we were unable to amplify RT-PCR products directly from the individuals materials or establish an immortalised cell line from the individual sample. We, therefore, performed exon trapping to test whether this variant altered splicing and generated a cryptic exon. Using the pSPL3 system,^31^ we observed that RT-PCR of the wildtype construct (pSPL3-EN1010-Wt) gave a single band with the expected exon 8 splicing, while the construct with the c.839+1309T>G variant (pSPL3-EN1010-Mut) gave an RT-PCR product with a 171 bp insertion following exon 8 (r.839_840ins171) (figure 3). The inserted 171 bp corresponded to c.893+1139 to c.893+1309. This insertion is predicted to cause premature termination of the WRN protein at position 280 (p.Arg280SerfsTer9).

### T-LRS identified a previously unknown large deletion and missense variants

T-LRS of individual PD1010 revealed a 338 715 bp deletion that began within *WRN* and included exons 25 through 35 (figures 1B and 2). PCR and Sanger sequencing of the predicted junction confirmed the deletion as chr8:g.31,135,822_31,474,535delinsTCT (online supplemental figure 2). Encouraged by the efficacy of T-LRS, two archived cases, individuals WV and FES, were then re-examined.^33^ Western blot analysis of these cases showed no WRN protein, and RT-PCR in the mid-1990s revealed a single heterozygous variant in both cases.^19^ T-LRS identified a heterozygous pathogenic coding variant, c.3961C>T, p.Arg1321*, in exon 33 of individual WV, and c.2103_2104delAC, p.Leu702fs, in exon 19 of individual FES (figure 2). In all three cases, phasing predicted that the previously known pathogenic variant was on a different haplotype than the newly identified second variant. That we identified variants in coding sequence in these cases was not surprising as they were enrolled in the registry prior to the availability of PCR and Sanger sequencing.

### T-LRS failed to identify a pathogenic variant in a case with known exon skipping

Individual SILV1010, a man from the USA whose presentation and phenotype is consistent with possible WS, was found to have a pathogenic heterozygous c.2665C>T, p.Arg889* in exon 22. Western blot analysis on lymphoblastoid cells demonstrated 1% WRN protein expression, consistent with WS. RT-PCR of material from a LCL derived from the affected individual showed heterozygous skipping of exon 20 (r.2274_2448del175) (figure 1C).^2^ T-LRS of DNA from this individual detected the previously identified c.2665C>T, which was predicted to be on haplotype 1 (figure 1D), and a second variant (c.1269+36A>G) located in the ninth intron predicted by SpliceAI to affect splicing by creating a new donor site with a score of 0.45 and loss of a donor site at c.1269 with a score of 0.12. However, we did not detect splice alteration involving exon 9, intron 9 or exon 10 in LCLs from the affected individual, nor did we find pathological significance linked to this variant in literature or databases. Thus, we felt this variant did not explain skipping of exon 20 in this individual. Because exon 20 contained a heterozygous c.2361G>T, we performed WGS of RT-PCR products on an ONT Flongle and found that approximately half of reads skipped exon 20 (online supplemental figure 3) and that those RT-PCR products which contained exon 20 were homozygous for the reference allele G and did not contain the T allele that was predicted to be located on haplotype 2 (figure 1D). A single SNV on haplotype 2 with an allele frequency <1% and absent from population databases was found in either intron 19 or 20 (c.2273+155A>G). Unfortunately, this variant is not computationally predicted to alter splicing. Thus, sequencing of RT-PCR products did not reveal a clear potentially pathogenic variant, and this case remains unsolved at the molecular level.

## Discussion

Here, we show that computational selection of specific genomic regions for sequencing using adaptive sampling on the ONT platform can be used to identify disease-causing variants in a cohort of individuals with WS. Different approaches for T-LRS have been described using both PCR and CRISPR/Cas-based methods.^34–36^ PCR-based methods allow for targeting by overenrichment of target regions but remove epigenetic information from the target and are limited by the length of the fragment that can be reliably amplified by PCR. CRISPR/Cas-based methods work by first dephosphorylation of genomic DNA, then exposing new phosphorylation sites with targeted double-stranded breaks where sequencing libraries then anneal. The advantage of the CRISPR/Cas method is that epigenetic information is preserved while segments up to about 200 kbp can be targeted. The drawback is that multiple regions must be targeted if one wants to target several megabase pairs of genomic space, and recovery of large fragments may require gel-based separation of cut DNA fragments. In both cases, fragments can be sequenced on either an ONT or PacBio platform.

Recently, adaptive sampling on the ONT platform was shown to be an effective method to evaluate known structural variants and missing variant cases.^18^ This method works by computationally selecting DNA molecules for sequencing.^20^ There are several advantages of adaptive sampling over CRISPR/Cas and PCR-based methods, including broader flexibility in selecting target sites, ability to target a larger amount of genomic space and simpler library preparation steps without the need for DNA fragment isolation by pulse-field gel if large segments are targeted. However, ONT sequencing has a higher per-read error rate than PacBio high-fidelity (HiFi) sequencing because each DNA molecule is read only one time by ONT instead of multiple times as with PacBio HiFi.^37^ This error is most apparent in homopolymers, meaning that certain classes of variants are more likely to be missed by ONT sequencing.

Using adaptive sampling, we identified a second pathogenic variant (including missense, deep intronic splice and structural variants) in eight of nine individuals clinically diagnosed with WS and a single known pathogenic variant identified by prior testing. While all eight variants identified using T-LRS would likely also have been identified by short-read genome sequencing (WGS) and focused analysis of *WRN* there may be a cost advantage of T-LRS over short-read WGS in missing variant cases such as these. Each individual was sequenced on a single ONT R9.4.1 flowcell after a single library preparation, representing a materials cost of approximately US$600 per sample which is cost competitive with short-read WGS today. Exome sequencing, on the other hand, would likely identify the two missense variants but may have difficulty identifying the large deletion in individual PD1010 and would be unable to identify deep intronic variants because those regions are not typically targeted for capture or enrichment.^38 39^

The major advantage of our approach over short-read WGS is the ability to resolve repetitive regions and phase samples for which parental samples may not be available. *WRN* contains two 1400 bp segmental duplications involving exons 10 and 11 as well as surrounding intronic sequence that are approximately 95% similar. Prior work using PCR to identify pathogenic indels and nonsense variants in exons 10 and 11 depended on a single-nucleotide difference between these two regions to design region-specific primers.^2^ Thus, there is a technical advantage of T-LRS over short-read WGS to cover such regions.^40^ Furthermore, unlike short-read WGS, T-LRS is able to more easily phase variants in the absence of parental samples for segregation, representing an additional advantage of T-LRS over short-read WGS. Thus, a strong argument exists for T-LRS the next best test for individuals with non-diagnostic clinical testing and either a compelling phenotype for a specific disorder or a single known variant in a gene associated with the suspected disorder.

That four of the missing variants identified in our study were splice variants demonstrate how difficult these can be to detect in the laboratory. Here, in silico analysis allowed us to identify candidate splice variants and allowed for targeted evaluation in our unsolved cases. Interestingly, the highest reported heterozygote frequency for a pathogenic WS variant is a deep intronic splice variant, c.2089-3024A>G, r.2089_2273del85 found in approximately 1:120 individuals in Sardinia.^13 19^

Despite advances in the ability of computational prediction algorithms to identify intronic variants that may alter splicing, it is still necessary to confirm the pathogenicity of these variants in the laboratory. When material from an affected individual is unavailable, CRISPR may be used to generate a cell line containing the candidate variants. Intronic regions, however, generally contain repetitive sequence that makes CRISPR mutagenesis more challenging. Therefore, in this case we used exon trapping as an alternative approach to validate the candidate variant identified in individual EN1010.^31 32^ Although exon trapping has been widely used for this purpose, there is a formal possibility that natural introns and exons may have an unknown influence on cryptic splicing.

Worldwide, WS is thought to be clinically underdiagnosed because many of the symptoms are relatively non-specific and may be viewed as common age-related diseases. We believe that additional genetic testing, including long-read sequencing, will lead to an increased rate of diagnosis for individuals with WS. This increased diagnostic rate and awareness will help drive interest in research and development of therapeutics, thereby benefiting all individuals affected by this disorder.

10.1136/jmedgenet-2022-108485.supp2 Supplementary data

## Data Availability

Data are available on reasonable request. Data that support the findings of this study are available on request from the corresponding authors.
