# Supplementary material for: Targeted long-read sequencing identifies missing pathogenic variants in unsolved Werner syndrome cases
Source: J Med Genet. 2022 May 9;59(11):1087–94. Online ahead of print. doi: 10.1136/jmedgenet-2022-108485 (PMC9613861; doi:10.1136/jmedgenet-2022-108485)
Supplement: Supplementary data [file jmedgenet-2022-108485supp001.pdf]

SUPPLEMENTARY MATERIALS

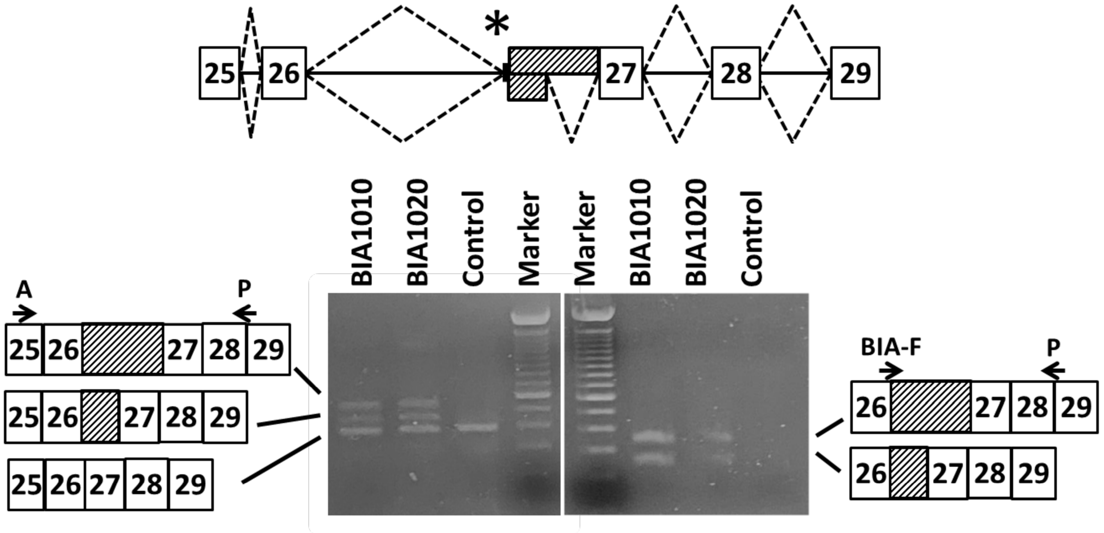

Supplemental Figure 1. A splice variant results in a cryptic exon within *WRN* in the BIA pedigree. RT-PCR of BIA1010 and BIA1020, both of whom are heterozygous for c.3234-170A>G in intron 26 (as shown by the asterisk (*) on top diagram), showed larger products using the primer design from exon 25 (A: 5'-ATCGGCAAGGATCAAACAGAGAG-3') and exons 28-29 (P:5'-TTCTGGTGACTGTACCATGATAC-3') (left panel). Using a primer designed from exon 26 and cryptic splice acceptor site (BIA-F 5'-GCTTCTGCCTAGACATCTTAAAGC-3'), two unique RT-PCR products were detected in both affected patients (right panel).

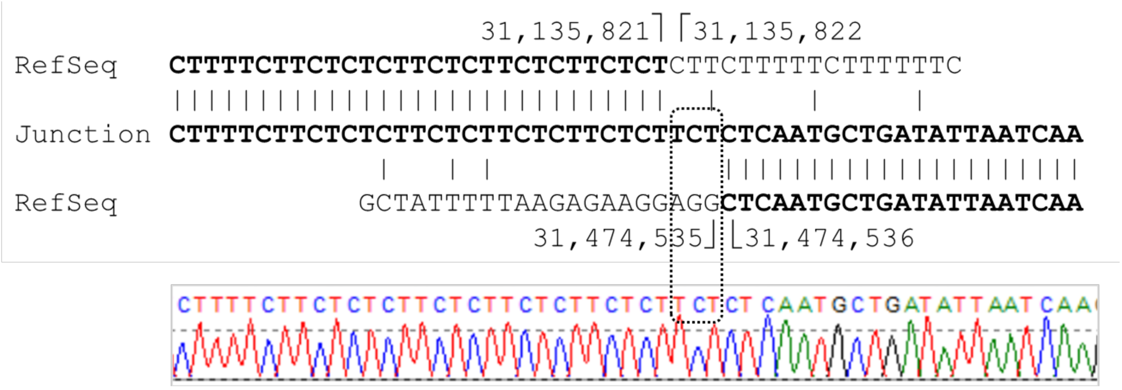

Supplemental Figure 2. Sanger sequencing of the large deletion identified in PD1010 (chr8:g.31,135,822_31,474,535delinsTCT, GRCh38). The patient's sequence (middle, described as "junction") was aligned to NC_000008.11. The dashed square shows the inserted TCT sequences. Bold letters show matched nucleotides.

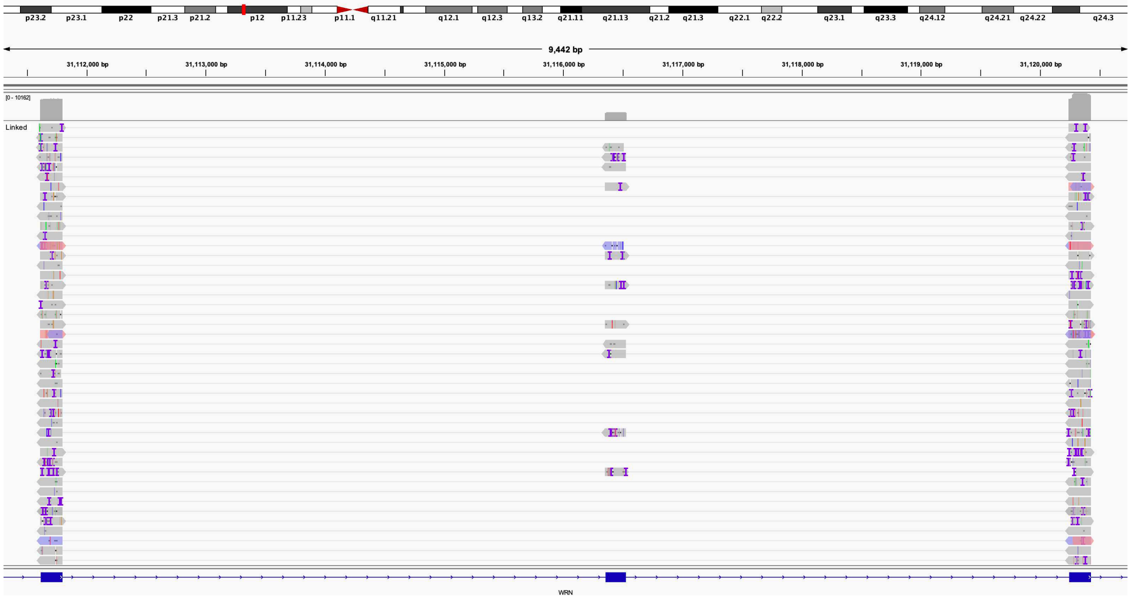

Supplemental Figure 3. IGV view of long-read whole-genome sequencing of rtPCR products from SILV1010. Exons 19–21 are shown at the bottom and reads are represented by gray boxes. Linked reads are shown by thin lines connecting the gray boxes over the exons. Note that coverage is approximately half for exon 20 when compared to exons 19 and 21. Exon skipping of 20 was known in this sample.

**Sample**	**Estimated coverage of target region**
WV	28.3
BIA1010	n/a
BIA1020	25.6
PD1010	18.4
CB4	13.3
CB6	20.2
FES	15.4
SILV1010	22.9
EN1010	14.3

Supplemental Table 1. Estimated coverage of each sample.

Gene or Region	Chromosome	Start	End
FMR1	X	147,400,000	148,500,000
WRN	8	29,900,000	33,700,000
COL1A1	17	49,650,000	50,750,000

Supplemental Table 2. Regions targeted for adaptive sampling on the Oxford Nanopore Technologies (ONT) platform using GRCh38 coordinates. Target region is WRN, while control regions are FMR1 and COL1A1.

Registry	Variant	CADD score	SpliceAI prediction	SpliceAI score
BIA1010, BIA1020	c.3234-170A>G	9.556	Acceptor site gain	0.32
CB4, CB6	c.1982-297A>G	11.89	Donor site gain	0.84
EN1010	c.839+1309T>G	9.358	Donor site gain	0.73
WV	c.361C>T, p.R1321*	44	NA	NA

Supplemental Table 3. CADD and SpliceAI scores for single nucleotide variants identified in this study. NA, not applicable.

BMJ Publishing Group Limited (BMJ) disclaims all liability and responsibility arising from any reliance placed on this supplemental material which has been supplied by the author(s)

Supplemental File 1. Sequences of the intronic variants and activated splice sites found in this study.
