# Supplementary material for: Targeted long-read sequencing identifies missing pathogenic variants in unsolved Werner syndrome cases
Source: J Med Genet. 2022 May 9;59(11):1087–94. Online ahead of print. doi: 10.1136/jmedgenet-2022-108485 (PMC9613861; doi:10.1136/jmedgenet-2022-108485)
Supplement: Supplementary data [file jmedgenet-2022-108485supp002.pdf]

Intronic variants (red), activated splice sites (blue) and region of cryptic insertion (gray) are shown with respect to WRN exons (yellow). See text for details.

c.839+1309T>G (Registry#EN1010)

```
    42841 ttaatgaagt ggctaaatga atatctctgc tttgtggttt gaaaattaat attgattttt
    42901 tttcccccta gaggaagaaa tcctacttag cgacatgaac aaacagttga cttcaatctc
E8  42961 tgaggaagtg atggatctgg ctaagcatct tcctcatgct ttcagtaaat tggaaaaccc
    43021 acggaggtta aatattacct tttttttttt taacttaaat caattctgtt tattttttta
    43081 tcacattttc ctatatgtga agaatactat tcattaaggc tgactcatag aaatttgctt
    43141 gaatacacac acacagactg atgcataaat ttgagaaata tctttccagt tgttgaatag
    43201 tgaatccaaa ggtttaagcc ttttctgtct gctttgtagg atgttttatg aaacaaagga
    43261 atagaaattg tattcatatt atacattttg aaattatatc tatattatat tttttgtcaa
    43321 agcaaaataa attatacatt aaggaaaata tttaagattc aaagttgtca ttttctttct
    43381 gttaatgagt atctttgatt taataacatt tatagtttgg tcacttgaaa tctatagtga
    43441 ccaaaagcaa tgtcatattt agtagcctca tttctgttac tatctgatta ttacttaaga
    43501 attctactta attaatagat tattagaaca aaaggcattc caattttaga gattctttta
    43561 tggttaaagc actaggtaac ttaaaaaatt tggatcaaat gttatacaga ttaatagacc
    43621 ttggaataca ggggctatgt ctttttggaa tcagaccttg tcctggcctt aattgcagga
    43681 tgacttcctt attaaatctc taaaagcagg aatgctctgg aacaatgaac aattagaatg
    43741 tggtaatgta attttgggcc aaaaaatact ttgtagaaat gtgattctct tacttggaat
    43801 agaaagaatt ctgactcgaa aaagtgcagt taatgtatgc ttttctgggt aagatttgtg
    43861 atagattttg attgagattt aaaaccatga tatataaatt tagctttttt gtcaaagtaa
    43921 tttgccaccc tagaatttgt gttggtaatt tttaatgcaa cttggacaat ttctgaaaga
    43981 tgtaactttc ttttttttga gacgagtctc gctttgttgc ccaggctgga gtgcagtgac
    44041 atgatctcag gtcactgcaa gctccgcctc ccgggttcac accattatcc tgcctcagcc
    44101 tcccgagtag ctgggactac cggcgcccgc caccccacct ggctaatttt tttttatttt
    44161 ttagtagaga cggggtttca ccgtgttagc caggatcgtc tcgatctccg gacctcgtga
    44221 tctgcccgcc tcagcctccc aaagtgctgg gattacaggc gttagccacc gcgcccagcc
    44281 tgaaagatga actttaaaat ggggaaggat actagtaaag ccatttctta ctcatgtata
    44341 catttttccc tccatgaaat tagagcagcc accttttaca tgacttttac atgcatttca
    44401 gatttttgta ctagcaatta aaatatctac tggatgtaaa tcacatgtgc ttaatgcttt
    44461 gcttcataat atctgttgaa gtcaattaaa atatagagac agatctttaa attgaacatg
    44521 ttttatttgg gaagcaagaa ttaaatttga ggcatataca cagactgggt gtccttggta
    44581 tctctgaaga acaaagagaa gtttggaggt tatagaaaaa ggaaaatgtg acattgtttt
    44641 tccagaaagt tctttggcac tagtaaattt tggggagttg gaagctctga tgggttagtg
    44701 actgtggtgg gtaaaactag tcttagagtc acaacaggtt gtttcagtat ctattagata
    44761 aaacaggttt taggttacaa taggcagttt cagcagctag gcttgcagaa aattacattc
    44821 ttagagtaat gttatgtgcc ttgagtgctt ttttcccctt ggcccttgac tctgatttag
    44881 ttgggtatga caagaattat caatttatat aattactttt tacatattga agctgaatag
    44941 gttttcttaa aacttggaaa tgggaaatac tgggtcttgt gaagttttag tgtgaaaacg
    45001 ataactagtc tcttatttca tgctctttgt aacaagtcta cagtaatacc taatgtatct
    45061 agatgacacc tcagaaccag gagggaacaa aaaagacctc tgagcagtca aaataatgct
    45121 ataagcatgt attccagctt actttcggca tttctgtgga gtgcaggagg ccacattttg
    45181 ttcatagatc cagtttgaca agcttgggta ctgagttaaa gtgaaaatgg tgtaaataag
    45241 taatcatatt cacatgacag taggtggaag tgacagctga caatctgaaa gagtggacaa
    45301 aactataaaa attagaacta agaactctta aaatagaacg gtttgaagtt tgttacagaa
    45361 gggagcagct ttatgcttca gtggactctg aaggtattgg cagtgtatag tactgcttag
    45421 tcattgattt aaattcggta tatatctctt ctattgctgt catgtacttg acattctgct
    45481 gggtgctaag gctgtctgca ggctactaat atgcataata acaatcctga aaaagaagga
    45541 aaagtatact gtgccttact gaagaaggca ggacagccgt tggtatttgt tagagtaaat
    45601 attagttatc ttacataaag catgttatat tccctgatgt cagggcctga taaatgagcc
    45661 cttacactct tgatagtcaa tctgtgccac tgttgctgtg tttgtatgtt acaccatttc
    45721 ctgtccatgt aacagttaaa ctgtatcaca tatctgttcc tactaaacta tttgcatata
    45781 attttaacat ttgagtggct gtatataaag ctaaaagttc tttataagat ttatagcatc
    45841 ttagaatgtt taaattagat aacattagtg tgcttttagt ttttgaattt aattttgctc
    45901 ttgattttgt ttgtgttaat gtagactttg tctacgtaag cttgagccca actttggagt
    45961 ggtgttttca gtgatggaag taactggaaa taactcatga aaccacttag tacttttgat
    46021 agtttattcc catgaaggat caaattttta cttgagtcat taatataaaa atttaaaaga
    46081 catgtcagat tttattaagc atggatttta aatatataaa tattagaaga cctaaacaaa
    46141 atcgctaatg aaagagtaaa acatgtccct gcctatcata ctcccttcac tttcactgat
    46201 ttctgctttt atgaagtact ttaataagct ctacaacaaa taaacaagac attgtgcctc
    46261 attttacact ctagcagtgg ttttccattc caactaacta gattgggttt ataatatagt
```

```
     46321 catttaaact tacttagtta aaatgttata cattagcaac tgcaattaag gagctaagaa
     46381 attcagctcg tatttattta tatgcatctt atccatagtt tctcacttgt tatatttgcc
     46441 tactgatggc aaattgattt tttctgttcc aaaaatgtag tgttttgaga tttttggaga
     46501 tgcaaaacag catcattttc aaattagatc acaaacaatc ttacttgttg cagttgagac
     46561 tagtaattga tcagtgaggt gaaaaaatta ggcaaaacgg tatcatcaaa attttctttc
     46621 tttttttttt tgaaacagag tattgctctt gtcgcccagg ctggagtgca atggcacgat
     46681 ctcagctcag ctcactgggg cccctaccta cgggattcaa gggattctcc tgcctcagcc
     46741 tcctgagtag ctgagattac aggcgcatgc caccaagccc agctaatttt tgtattttta
     46801 gtagagacag ggttttgcca tgttggccag gctggtctgg aacttcagac ctcaagtgat
     46861 tcacctgcct cagcctccca aagtgctggg attacaggca tgaaccaccg tgcccgcccc
     46921 aaattttctt ttaacttaaa gtgttaatgt ttgtttgttc ccaatatttt tatatatgtc
     46981 aaggcttttt cattatatac aggccagctc tttggagttc tgcatagtgt ttcttgttta
     47041 aaccacacct ataatgtacc atctacaatt tccaattctt taagacagag tgaaaaatta
     47101 agacacctga agcaatctga gtgcggagtc ctaggcatta tgattttcct tgacactttt
     47161 agggttcttt tatcacagtt tatcaaacac cttttttttt ttaacaaaaa acaaatttac
     47221 aaaatctttc catgtattca acttagaatt taatgaaata gggttttttt tttttggtg
     47281 gtcatgtatc atcaactttc tttacattta attaaattaa tcaacaaaaa acatatagtt
     47341 gacataaaca aatttgggac caagcactac tgactcttag taaacataca agtcaactgt
     47401 tggtgcagaa gtgccttgat gctagagagt agcctgcaag ctgtaattat atatgggcat
     47461 tagggaatga agaacagcct taatactatt gatcttttaa gtgaaggtca gcttagaaag
     47521 cttttacttg ttaaaaagct tcacagtttg tccttgtagt taatgcaatt gaagttgaat
     47581 taatctttct taattttttt tttaggtttt ctatcttact aaaggatatt tcagaaaatc
     47641 tatattcact gaggaggatg ataattgggt ctactaacat tgagactgaa ctgaggccca
     47701 gcaataattt aaacttatta tcctttgaag attcaactac tgggggagta caacagaaac
     47761 aaattagaga acatgaagtt ttaattcacg ttgaagatga aacatgggac ccaacacttg
E9   47821 atcatttagc taaacatgat ggagaagatg tacttggaaa taaagtggaa cgaaaagaag
     47881 atggatttga agatggagta gaagacaaca aattgaaaga gaatatggaa agagcttgtt
     47941 tgatgtcgtt agatattaca gaacatgaac tccaaatttt ggaacagcag tctcaggaag
     48001 aatatcttag tgatattgct tataaatcta ctgaggtact aaataaagag gaagcacatt
     48061 tttagttatt agtaggttct ggcagacttt attcccgtaa agagacagat agtaaatatt
```

c.1982-297A>G (Registry#CB4 and CB6)

```
     63421 tccttgagat gattgttttc tttattgtta atatgtttcc cttcctgttt tttttttttt
     63481 ctttttttctt ttgtttgttt ttacagaggt aaataccgga ttgtatacgt aactccagaa
E17  63541 tactgttcag gtaacatggg cctgctccag caacttgagg ctgatattgg taagtgataa
     63601 agaaagatct ctgtaaatac ttactgagtt aatatttaaa gttaaaccta tggatggaca
     63661 ctggatttca cttctgttaa agtttatttc aaacattact tcctccagga aatctctgac
     63721 tctctaactt gctgtttcac ctatcttatc ccatgatact ctataattcc ttttgctata
     63781 gaaattaggc tctgttatca taattacctg tttatatatc tgttttcttc tgctaggcta
     63841 taaacttttc aagggaaaaa tatcactttt taaaatttat gtttccccag tgcagtgctt
     63901 ggcatatagg cacttaaaca tattgttgaa tgaatgttga atcctaggtt ctgtgaaatt
     63961 ttaatttact tgtttttcat gttctgtatc taaattaagt cagtgtagtg gttttgtacc
     64021 ctctttgttc ataagaatca cttttcaaac tttaaaaaaa atgtaggtaa ccaacaccca
     64081 actctgaaaa ttctgattta acaggcctgg gttggcacct ggcattggtg gtttctcaca
     64141 ggtgattctg atatatagcc cgggttggaa accacagtac ctttgattag ttgccactag
     64201 tgagtatgat ttattacaaa aaccaaaaac ttagataagt cacagtttat ttcctagaac
     64261 gtgtaaatat agaattagtc aaaagctgat agcacttttt ttgtacttaa aaatatttta
     64321 agcctcagta tagtgagacc tcatctctac aaataatttt ttaaaaaatt ggccgagcct
     64381 ggtggtgcat gcccatagtc caagctactt gggaggctga ggtaggagga ttgtttgagc
     64441 ctgggagatg aaggctgcgg tgagccaaga tcatgctact gcactccatc ctgggcaaca
     64501 gagcgagacc atgcctcaaa aaaaattttt ttttcttctt ggtggttagg cttaactatc
     64561 caagaattgc aatcatggct tctattgcaa atgcccctat ttcccacctt cagttggcct
     64621 aaccaaattt aatttttcaa tatcatatga tctttttgta taattatgta ttcaaataat
     64681 tccagatagg ttaggttttc gaattgcaac gtctcattta atggttactt tcatataact
     64741 gtaggaaagc atttgataat ttttccaaca atgtaatgaa aacaaacttg tatctttttt
     64801 attatgtgtt aaaaccaaca aaagaatgag ggcagcaatt aacatttcat taaatatatt
     64861 agcaatgagt acatttatga gctcttaaac tcccttattg acatactgta ttttttgcct
     64921 ttaatggaca atgaatttga tatgtttcct aataggttgt atgaagttat tgtgaatatt
     64981 tcactttgga ttaagaataa ctaccctata tataaatttc atggttgtat tgcaaaagct
     65041 ggggtctatg tccatagtgg tttgggtacg tttttactat ttgctttgtt tctgtgatag
     65101 tgttaaatga ataataagct taagtattta agcaaatact taatacttct gacaagtagt
     65161 tagaactctc tatttttgtt tttttgtaat ggatgagtta tggacactgc aattattaga
```

```
    65221 gcagcgtact ttgcttatga tggatgaaaa tgttctctgg ttgactcagt gttgaataaa
    65281 aagggataca tgaaataaaa attatttttg aaatattttc ctttgccttt tgttgcggga
    65341 aattgtacaa tgcttgcaaa aatgaatata cgcaggatat tatttgtaca gcagtctgac
    65401 atttagctaa tcagattcct catttttagg taaatgttaa attacttaag tagtctggat
    65461 ttttaacaga aacagcaaac atagttttat agggaggctc cttaccaacc agacttttaa
    65521 cttaagtgaa tgttggcaaa agcttgtcta aactgatttc cagggataag gtttagctcc
    65581 attaaaagct agtttgtcaa acctgtaact aaagcttttt aaaaaatgaa agttttaaaa
    65641 gagaccatca ttctgtttta acctatttaa tttaaaatct gggctgaagt aggagtattg
    65701 cttgaaccca ggagttcgag accagcctgg gcaacacagt gagaccccat ctctgaaaaa
    65761 aaaaaatgag ctgagcatgg tacctgttac agtgctagtt atgaaagaaa gaaagagaga
    65821 gagagagaag gaaggaaggg agaaaggaag gaaggggctg ggagtggtgg ctcacacctg
    65881 taatcccagc actttgggag gccgaggcgg gtggatcatg aggtcaggag atcgagacca
    65941 tcctggcgaa catggtgaaa ccctgtctct actaaaaata caaaaagaaa aaattagccg
    66001 ggcatggtgg tgggtgcctg tagtcccagc tactggggag ggtaaggcag gagaatggca
    66061 tgaacccggg aggcagagct tgcagtgagc cgagattgcg ccactgcact ttagcctaag
    66121 aaaaggaagg aaggaaggaa ggaaggaagg aaggaaaaat cagtcactca ctcaggttta
    66181 gtttcaaaag ccaaaacagt gataatattg aatttttttcc agccactcag tagtacccag
    66241 agaattttga tacaaataac tgactttgac cccatgttta gtaggttttt tttttgtttg
    66301 tttgtttgtt tttttttttt gtctttcttc tccacactct cttaggactg ttgtagcttg
    66361 taatgttagt gaattgtaat tgtttatttg catgtctgtc tcctctttta gaattgtgaa
    66421 ctcctcaggt catggattgt attttattag tctttttatt cctagaaaag tgtttggata
    66481 ataatagata tttattaacc ataataggag tagtagcatt tttgtgtgtg tgtgccaggc
    66541 cctattttag cattttatgt gaatgatata atttaatttt tagagcatct agaaaatgtt
    66601 aggtaccatt attatccaca ttttacttgt gagaggactg agacttggaa agcttaagta
    66661 acttgcttaa ggtaatacac atggcagatg gtggagccag gttctgcatc tgggcagtct
    66721 gactttagag ctttttaatc atcctcctcc tagtgtccga acattattaa tgctcagaga
    66781 agtatcaaag aatgatgata ctcattgagg gaaaaaatta ttcttaagta tcaatttgtt
    66841 aattgaggac aaaagcacta ctgttactac tgtgaaaaat ctgaaaacat tgtcacggat
    66901 gattttacaa taacatcctt ttctgacatg cattacaaca cactattgat tccgtttcaa
    66961 tgtttgtgtc tggtatgggg gcggagtaga gaggctacta caggaagaaa gaacaaataa
    67021 catttcttct tactactttg tgcatcatgt gatttagctc ctcccatacc attttacatt
    67081 agaaaatgtg gctgtcctcc tgtgctctag tactggcatt tcagaggact ctttgcaata
    67141 tgatatgtga tcaatcatgg atggcgatgc agttaatttc agtagaaatt tgctgactct
    67201 ttttatctct tttaatagct aatgcatgag ataaaattga aggtattacc aaggagaaat
    67261 gcaggtggat agttttggag gcaaagtcac agtaagcaat tcatctctcc aaggaatagt
    67321 gatgaaagct gagatccaat aaatgtcttg tttggattaa tacataaata tttcctttta
    67381 aaattcttaa ggcttttatt catatatctc ccctctccct atttccaata atgatttttg
    67441 gtgttaatat gatgatattt atgcttctgt ttttatagga accaaatact ttgatttggt
    67501 tatttattct cctttggaga tgtagatgag tttatttttt cctttcgagc tttatctttt
    67561 cctttatgtg tttttctttt tttacaggta tcacgctcat tgctgtggat gaggctcact
E18 67621 gtatttctga gtgggggcat gattttaggg attcattcag gaagttgggc tccctaaaga
    67681 cagcactgcc aatggtaagc tttgccaagt ctgatgtccc gaaattacat tcttaataag
    67741 gagagcattc aggattgggg agtggtaaag aagctgaaga cttcactata aaagagcaaa
```

c.3234-170A>G (Regitsry#BIA1010, BIA1020

```
   108361 ctgtttgact taattttgtt tcccactcca cattaaaaga tcctttttgc ttttaatagg
   108421 gtagaaattg gcttcataaa gctaatacag aatctcagag cctcatcctt caagctaatg
E26108481 aagaattgtg tccaaagaag ttgcttctgc ctaggttcat ttttcagttt ttttcttgta
   108541 acttctgcat tttttgttgc tatttatgtg attcaaatta taccagttta taggcctctc
   108601 acaagtaaaa tgaattacct gtttgttttt gtatgcctat tttagtcagt ttgggggaag
   108661 ggatctgtga ggaaaggata agtcatagag cacttttctt ttttaagaga cagagtctct
   108721 ctgtgttgct caagctggag tgcagtggtg cgatcatagc ttactgcagc ctcgatctcg
   108781 tgggcccaag taatcctcag ccacctgagt agatgggact acagacatgc actactatgc
   108841 ccagctaata tattttaatt ttttgtatag agacagggtc ttctagtgct tcctaggctg
   108901 gtcttgaact cctgagctca agtgatcctc ctgcctcagc ctcccaaact actgggatta
   108961 caggcatgat ccaccgctcc cagccagaac attttcttgg ttgatgggaa gtagctgacc
   109021 atggtattta gaaaacttct ttctcatcga ttaaagaagc agtactgaaa tcaatgcgga
   109081 ggaatccata tatcatattt acttctggtg tgtagaagtg gaaagggaat acatttgttg
   109141 cttacttttt tgtaccttta catgtgattg atcacttgtg agttttttct ttcaaacatc
   109201 ttaaagcttc cagagctttt tctagaaaaa aaaatcagtt ttaagaatca ccagttctaa
   109261 aagggtaata tcttattcat ctttctgaga atggagtatc atgattcatg aattagatac
   109321 ttgcatctta acatttgaaa taatttaatt ttattatttt ttagttcgaa aactgtatct
```

```
E27109381 tcgggcacca aagagcattg ttataatcaa gtaccagttg aattaagtac agagaagaag
   109441 gtttgtttta aagaaattgt tctgatttat ttcattcttt attgattcaa attctgttta
```
